# The biomechanical evaluation of metacarpal fractures fixation methods during finger movements: a finite element study

**DOI:** 10.3389/fbioe.2024.1457871

**Published:** 2024-09-05

**Authors:** Mingrui Liu, Lincong Luo, Tao Lin, Xiaoyu Lv, Manoj Kumar Vashisth, Jiaying Li, Jianlin Shen, Lin Xu, Wenhua Huang

**Affiliations:** ^1^ School of Basic Medicine, Dali University, Dali, Yunnan, China; ^2^ Yue Bei People’s Hospital Postdoctoral Innovation Practice Base, Southern Medical University, Guangzhou, China; ^3^ Guangdong Engineering Research Center for Translation of Medical 3D Printing Application, Guangdong Provincial Key Laboratory of Digital Medicine and Biomechanics, National Key Discipline of Human Anatomy, School of Basic Medical Sciences, Southern Medical University, Guangzhou, Guangdong Province, China; ^4^ Central Laboratory, Department of Orthopedics, Affiliated Hospital of Putian University, Putian, China; ^5^ School of Basic Medical Sciences, Southern Medical University, Guangzhou, China

**Keywords:** oblique metacarpal shaft fracture, finite element analysis, dorsal plate, intramedullary nail, Kirschner wire, screw

## Abstract

**Objective:**

This study used finite element analysis to simulate four commonly used fixation methods for metacarpal shaft oblique fractures during finger motion and evaluate their biomechanical performance. The aim was to provide evidence for clinically selecting the optimal fixation method, guiding early rehabilitation treatment, and reducing the risk of complications.

**Methods:**

Finite element analysis simulated dynamic proximal phalanx motion (60° flexion, 20° extension, 20° adduction, and 20° abduction). We analysed stress, displacement, and distributions for dorsal plates, intramedullary nails, Kirschner wire, and screw fixation methods.

**Results:**

At 60° of finger flexion and 20° of abduction, plate fixation demonstrated greater stability and minimal displacement, with a peak displacement of 0.19 mm; however, it showed higher stress levels in all motion states, increasing the risk of failure. The stability of the intramedullary nail was similar to that of the dorsal plate, with a maximum displacement difference of 0.04 mm, and it performed better than the dorsal plate during adduction of 20°. Kirschner wire showed the highest stress levels of 81.6 Mpa during finger flexion of 60°, indicating a greater risk of failure and unstable displacement. Screws had lower stress levels in all finger motion states, reducing the risk of failure, but had poorer stability. Stress and displacement distributions showed that the dorsal plate, intramedullary nail, and Kirschner wire mainly bore stress on the implants, concentrating near the fracture line and the proximal metacarpal. In contrast, the screws partially bore stress in the screw group. The anterior end of the metacarpal mainly hosted the maximum displacement.

**Conclusion:**

This study demonstrates that under simulated finger motion states, the dorsal plate fixation method provides the best stability in most cases, especially during finger flexion and abduction. However, high stress levels also indicate a higher risk of failure. The intramedullary nail is similar to the dorsal plate in stability and performs better in certain motion states. Kirschner wire exhibits the highest risk of failure during flexion. Although screws have poorer stability in some motion states, they offer a lower risk of failure. These findings provide important reference and surgical selection strategies for treating metacarpal fractures.

## 1 Introduction

The human hand represents one of the most intricate anatomical structures, comprising 27 bones and numerous muscles, tendons, and ligaments, enabling it to perform various complex and delicate movements (Moran, 1989). However, the hand is also a common site of injury in workplace accidents or traffic incidents, with metacarpal fractures being the most frequent type of hand fractures, accounting for 36%–42% of all hand fractures, with 58% occurring in the metacarpal shaft region, particularly the fourth metacarpal shaft (Xing and Tang, 2014; Dreyfuss et al., 2019). Orthopaedic surgeons usually categorise metacarpal fractures as transverse, oblique, spiral, or comminuted. Conservative treatment with cast immobilisation is suitable for stable and non-displaced fractures. However, oblique and spiral metacarpal fractures usually present with displacement and angulation deformities that interfere with the recovery of hand function, necessitating surgical fixation to reposition the fracture site and restore hand function (Greeven et al., 2016; Chiu et al., 2022). Solid fixation allows patients to engage in early range of motion activities, reducing the likelihood of stiffness in the metacarpophalangeal joints (Brewer et al., 2023).

Among the methods of fixation of metacarpal fractures, dorsal plates, intramedullary nails, Kirschner wire and screw fixation are widely used. However, experts still need to determine which fixation method yields optimal outcomes. Dorsal plate fixation provides greater stability but may lead to complications such as joint stiffness, tendon irritation, tendon rupture, and radial or ulnar nerve injury (Chiu et al., 2018; 2022). In contrast, intramedullary nail fixation offers the advantage of faster return to daily activities and work-related tasks (Beck et al., 2019). However, Ten Berg et al. (Ten Berg et al., 2013) found in a CT model of the metacarpal head that the use of intramedullary nails can damage articular cartilage at the metacarpal head, which may trigger the development of early osteoarthritis. Kirschner wire fixation is associated with lower costs and minimal trauma. However, Ridley et al. (2017) found that the likelihood of pin tract infection in metacarpal fractures treated with exposed Kirschner wires is twice that of buried Kirschner wire treatment. Moreover, using Kirschner wires for fixation also presents several drawbacks, such as local structural damage, wire displacement, and protrusion hindering early mobilization (Sharma et al., 2007; Wong et al., 2016). Compared to these methods, screw fixation is minimally invasive, cost-effective, and time-saving but lacks mechanical stability and presents technical challenges (Chiu et al., 2022).

Previous studies on metacarpal fractures have relied on animal or artificial bones due to difficulties obtaining fresh human metacarpals (Ridley et al., 2017; Oh et al., 2019; Labèr et al., 2020; Chiu et al., 2021). These methods offer advantages such as relatively low cost and easy accessibility. Additionally, they provide stable and controllable experimental conditions for anatomical and biomechanical studies. However, these methods also have limitations. For example, using cadaveric bones involves a limited sample size and may be influenced by bone quality. Although animal bones are more controllable, they are anatomically different from humans, so their findings are not entirely applicable to humans. Although artificial bones provide consistent experimental conditions and may differ in biomechanical properties from real bones, thus restricting accurate simulation of real-life scenarios. In contrast to the research methods, finite element analysis (FEA) offers significant advantages. Firstly, FEA can simulate based on real human anatomical structures, thus more accurately reflecting the situation of the biological system. Secondly, FEA can reflect the model’s displacement and internal stress information, providing precise quantitative and visual qualitative analyses with good repeatability and comparability while reducing experimental costs and risks (Lin et al., 2023; 2024).

Although many scholars have adopted FEA to simulate metacarpal fractures on computers, they usually investigate the biomechanical properties at rest by applying the corresponding external loads. They do not test the performance of different metacarpal fracture fixation methods under simulated finger motion. (Butz et al., 2012; Meng et al., 2013; Hutchison et al., 2022; Zhang et al., 2022).Thus, this study aims to utilize finite element analysis to simulate the dynamic processes of flexion by 60°, extension by 20°, adduction by 20°, and abduction by 20° of the proximal phalanx around the metacarpal head. The study investigates the biomechanical performance of four fixation methods—dorsal plate, intramedullary nail, crossed K-wire, and double screws—on oblique metacarpal shaft fractures. The study seeks to provide clinicians with more accurate treatment choices, guide early rehabilitation, and minimise risk of treatment complications, thereby improving patient outcomes.

## 2 Materials and methods

### 2.1 Three-dimensional modeling

**FIGURE 1:**
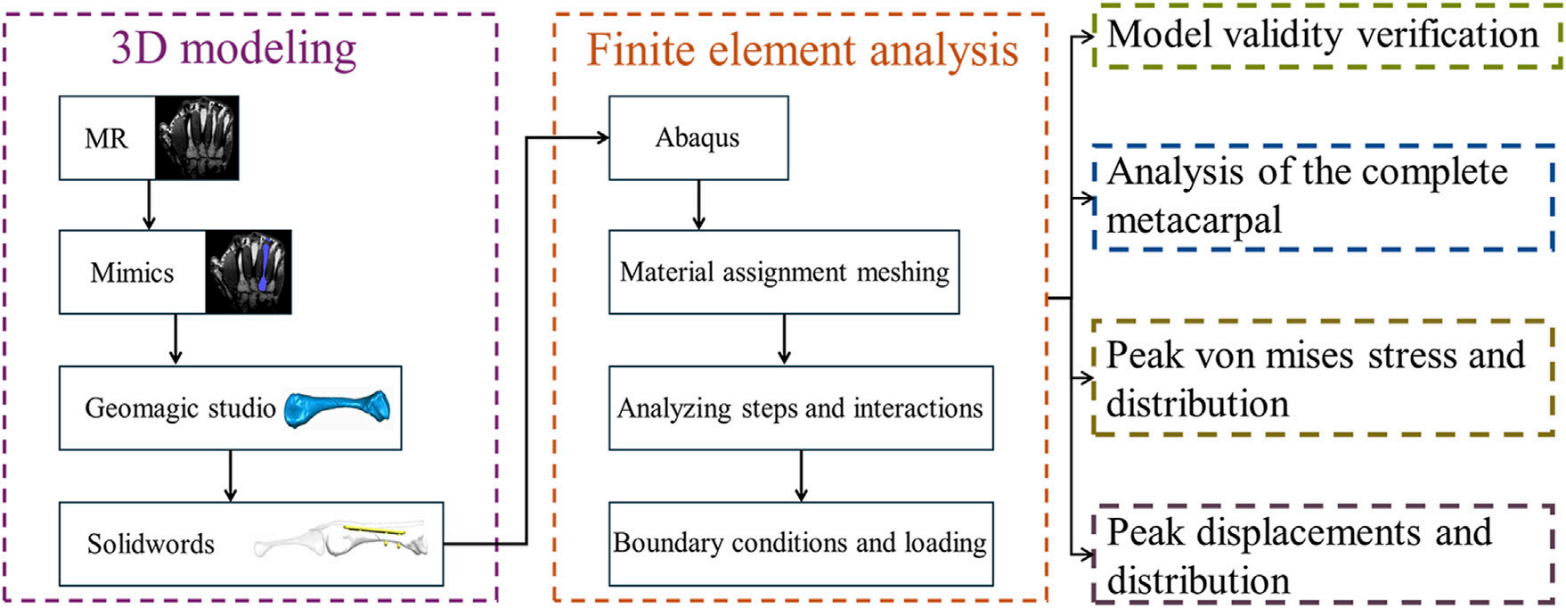
Modeling and analysis flowchart.

**FIGURE 2:**
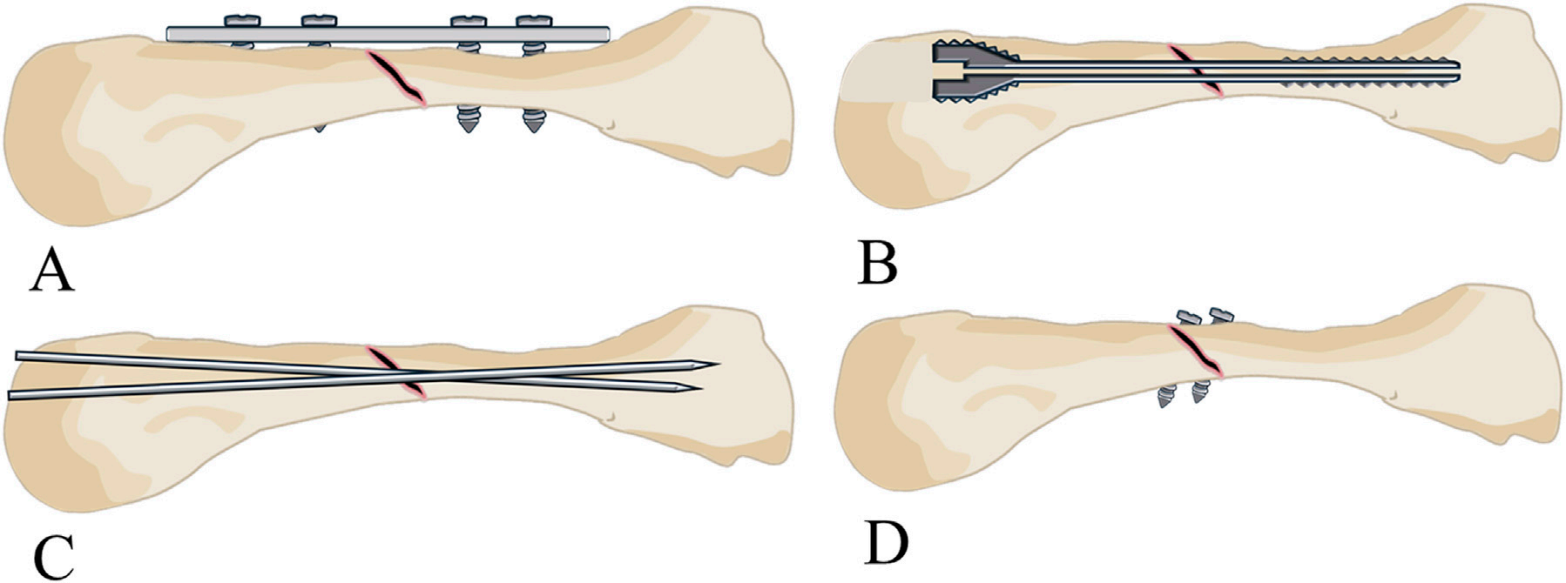
Establishment of the three-dimensional models. **(A**) Three-dimensional model of the fourth metacarpal shaft oblique fractures fixed with a dorsal plate. **(B)** Three-dimensional model of the fourth metacarpal shaft oblique fractures fixed with an intramedullary screw. **(C)** Three-dimensional model of the fourth metacarpal shaft oblique fractures fixed with two K-wires. **(D**) Three-dimensional model of the fourth metacarpal shaft oblique fractures fixed with two screws.

(Figure 1) depicts the modelling and analysis process. A 25-year-old healthy male volunteered for left palm scanning using magnetic resonance (MR) equipment (Siemens, Germany) with a scan thickness of 0.4 mm, generating DICOM format image data. We imported the MR data into Mimics 21.0 software (Materialise, Belgium), segmented the fourth metacarpal, proximal phalanx, and extensor tendon using appropriate grayscale values, and applied thresholds via region growing. We then used manual editing tools to remove redundant parts and supplement missing areas, establishing preliminary three-dimensional models we saved in STL format. Next, the generated STL files were imported into Geomagic Studio 2013 software (Geomagic, American) to remove surface protrusions, patch model surface holes, delete internal fragments, mesh re-meshing, and smoothing. To better simulate reality, we created a cylindrical medullary cavity with a diameter of 2.12 mm using boolean operations. We also introduced a 1 mm-wide gap 20 mm from the metacarpal head to simulate oblique metacarpal shaft fractures (fracture length less than twice the diameter of the shaft). We then employed contour line editing, surface patch construction, and precise surface lattice tools until achieving satisfactory results. We exported the models in STP solid file format. Finally, we used SolidWorks 2021 software (Dassault Systems SolidWorks Corporation, United States) to process the results obtained in the previous steps and save them in part format. The models of the dorsal plate (thickness 1.3 mm), intramedullary nail (diameter 3 mm), two Kirschner wires (diameter 1.5 mm each), and two screws (diameter 2.7 mm each) were assembled with the fourth metacarpal, proximal phalanx, and extensor tendon using the assembly module to obtain models suitable for further analysis (Figure 2).

### 2.2 Finite element analysis

#### 2.2.1 Material assignment, meshing and quality analysis

**FIGURE 3:**
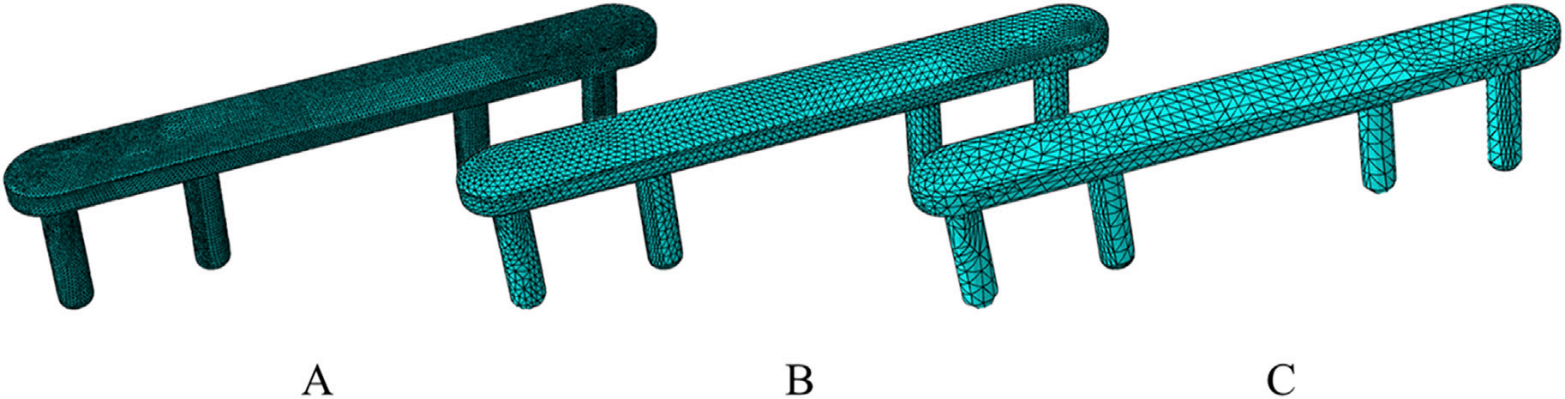
The fixtures utilize 3 different grid types. **(A)** Grid 1 has a cell size of 0.2 mm. **(B)** Grid 2 has a cell size of 0.5 mm. **(C)** Grid 3 has a cell size of 1 mm.

We conducted Finite Element Analysis (FEA) using Abaqus 2021 software (Dassault Systems, France) on the abovementioned combined models. First, we assigned material properties, performed meshing and mesh quality analysis. This study defined the material properties of the fourth metacarpal, proximal phalanx, extensor tendon, and implants as isotropic and homogeneous linear elastic materials. In order to determine the appropriate size of the unit used, good and accurate results are guaranteed at the lowest computational cost (Lostado Lorza et al., 2021). We carried out three sets of mesh convergence analysis with different densities. Specifically, we used densities of 2 mm, 1 mm, and 0.5 mm for the fourth metacarpal and proximal phalanx; 2.5 mm, 2 mm, and 1.5 mm for the extensor tendon; and 1 mm, 0.5 mm, and 0.2 mm for the implants (Figure 3). As the mesh density increased, the fluctuation in peak stress and displacement results of the metacarpal and implants gradually decreased and stabilized, with differences of less than 5%, indicating mesh convergence. We balanced accuracy, computational cost, and simulation efficiency. We selected the optimal mesh densities (1 mm for the fourth metacarpal, 1 mm for the proximal phalanx, 2 mm for the extensor tendon, and 0.5 mm for the implants). To ensure the shape and quality of the mesh itself, we performed a mesh quality check in verify mesh in the mesh module in Abaqus. The mesh size is reduced by reducing the mesh size in areas that are highlighted to improve the mesh quality. The material properties were consistent with previous work (Table 1) (Meng et al., 2013; Zhang et al., 2022).

**TABLE 1:** The finite element model’s material properties and element types.

	Young’s modulus (Mpa)	Poisson’s ratio	Type	Element
Proximal metacarpal	10,000	0.3	C3D10	32,510
Distal metacarpal	10,000	0.3	C3D10	24,067
Proximal phalanx	10,000	0.3	C3D10	17,909
Extensor tendon	276.48	0.4	C3D10	3,249
Dorsal plate	110,000	0.33	C3D10	22,740
Intramedullary screw	110,000	0.33	C3D10	12,141
K-wire	110,000	0.33	C3D10	2000
screw	110,000	0.33	C3D10	6,082

#### 2.2.2 Analysis steps and interaction settings

In the analysis steps, we employed the static general analysis module. We set the initial increment step to 0.01 and the maximum increment step to 0.1. In the interaction module, we applied contact settings to the metacarpal head and extensor tendon with a friction coefficient of 0.3, representing hard contact. Additionally, we used spring elements to connect the proximal phalanx, extensor tendon, and metacarpal. Specifically, 8 sets of springs with a stiffness of 6 N/mm were placed between the proximal phalanx and extensor tendon to simulate their connection. On the radial and ulnar sides, 1 set of springs, each with rotational stiffness of 55.4 N/rad and 65.3 N/rad, respectively (Werner et al., 2003), were set to simulate the action of collateral ligaments. Furthermore, we placed 4 springs with a stiffness of 0.5 N/mm between the metacarpal and extensor tendon. All implants and their corresponding implantation sites were set to a tied state to simulate adequate fixation.

#### 2.2.3 Boundary conditions and loading settings

**FIGURE 4:**
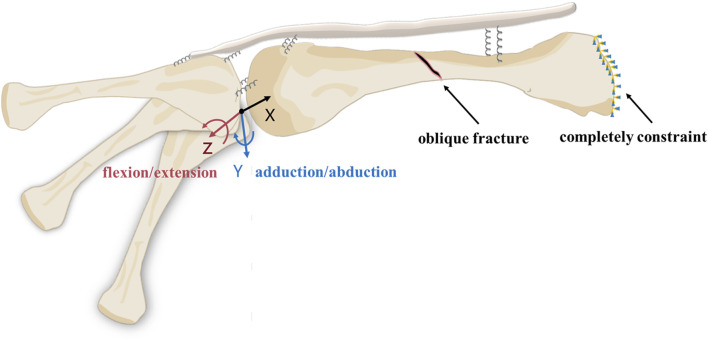
Loads and boundary conditions for models in finite element analysis. The proximal phalanx moves around the *Z* and *Y*-axes. The base of the metacarpal is set to be completely constraint.

The boundary conditions set up for the intact metacarpal group and the metacarpal fracture group were such that we fixed the basal surface of the metacarpal and limited all six degrees of freedom to zero using a constraint method (Figure 4).As the metacarpophalangeal joint involves a complex rolling and sliding motion during flexion and extension, the instantaneous axis of rotation varies. Therefore, the rotation of the proximal phalanx around the metacarpal head is not a simple rotation around the centre of a fitting sphere (Schultz et al., 1987; Tamai et al., 1988). To simulate the motion of the proximal phalanx around the metacarpal, we applied the following loading settings: First, we fitted spheres to the proximal phalanx base and metacarpal head. Then, a point was determined by connecting the centres of these two spheres and intersecting with the proximal end of the proximal phalanx. In order to avoid bone contact hindering the respective motion, we created a coordinate system below this point. In this coordinate system, the X-axis points to a reference point at the centre of the metacarpal head, while the *Z*-axis is aligned with the coronal axis of the proximal phalanx. With this setup, the proximal phalanx, which is considered as a rigid body, can be rotated around the *Z*-axis and *Y*-axis to achieve flexion, extension, adduction and abduction of the proximal phalanx.

## 3 Results

### 3.1 Model validity verification

**FIGURE 5:**
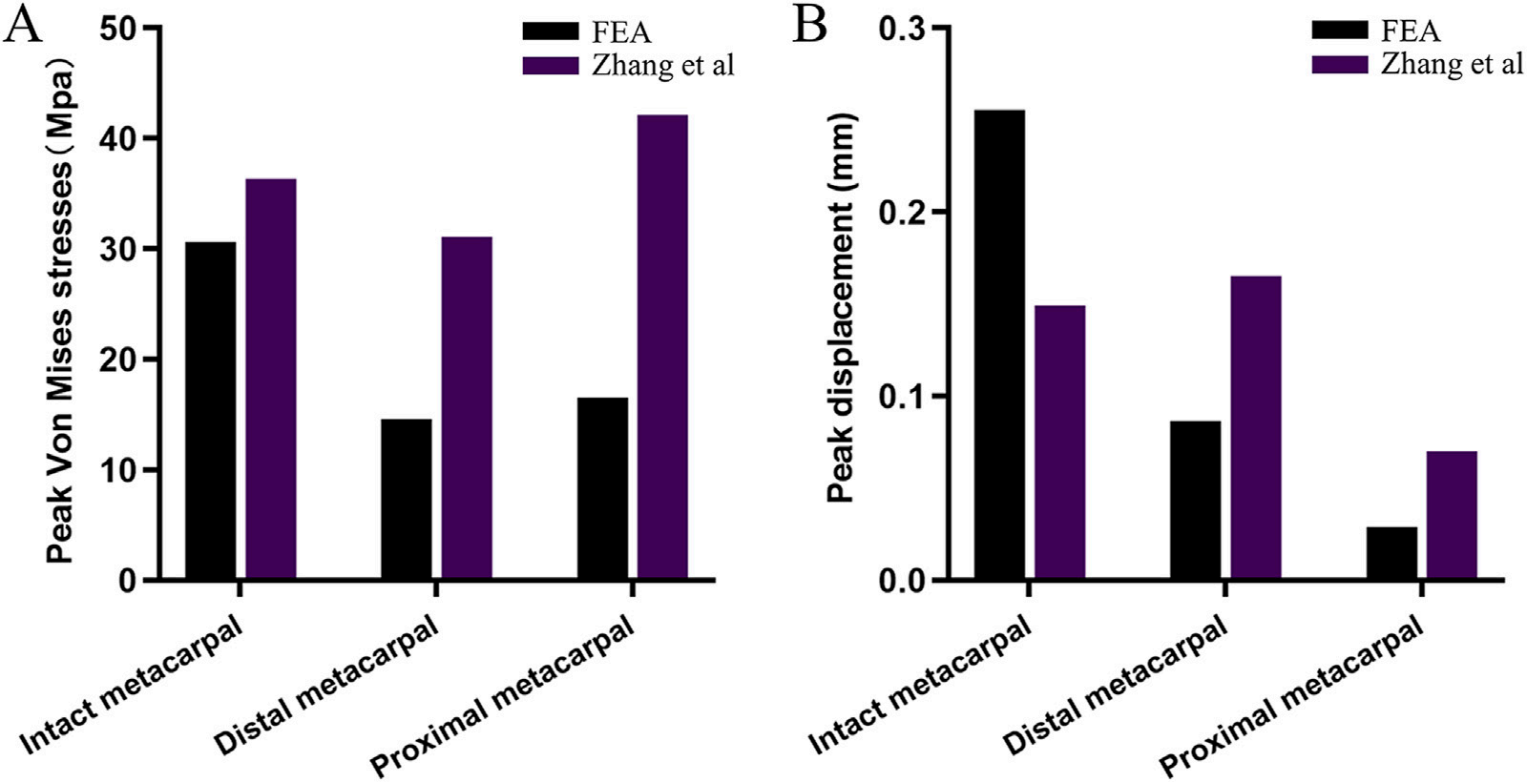
Comparison of **(A)** peak stress and **(B)** displacement results of finite element models of intact metacarpal and dorsal plane groups with existing results.

In our finite element model of the dorsal plate group, when the proximal phalanx flexed 60° around the metacarpal head, the maximum resultant force at the distal end of the metacarpal was 128.98 N, with a peak displacement of 0.277 mm. To validate the model’s effectiveness, Zhang et al. (2022) conducted mechanical tests on cadaveric metacarpal shaft oblique fractures fixed with plates containing different numbers of screws, subjected to loads ranging from 0 N to 150 N. The study results indicated that in the finite element model, under a load of 128.98 N applied to the distal end of the third metacarpal, the displacement ranged from 0.2 to 0.3 mm, similar to the results of our study model, thus validating the effectiveness of our model. Furthermore, we compared the peak stress and displacement of our study model under an approximately 100 N resultant force at the distal end of the metacarpal with Zhang et al.'s scenario of applying a 100 N force perpendicular to the long axis of the third metacarpal at its distal end, analysing the complete metacarpal, distal metacarpal, and proximal metacarpal (Figure 5). Mechanical behavior depends on the basic physiological characteristics of each individual, such as weight, height, age, etc (Somovilla-Gómez et al., 2020). For the finger model, small deviations are acceptable.

### 3.2 Analysis of the complete metacarpal

**FIGURE 6:**
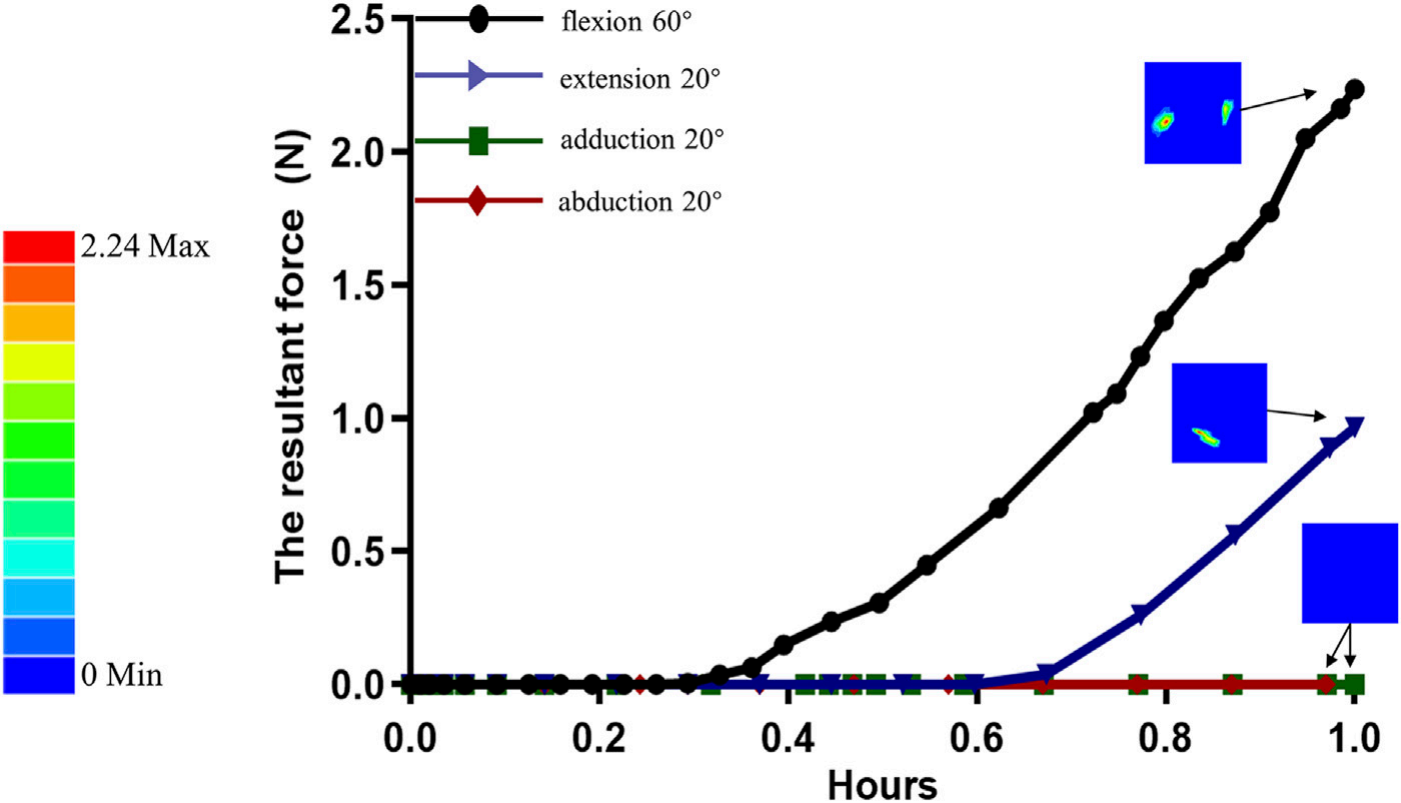
The variation curve of the resultant force acting on the metacarpal head due to the combined stress and friction from the extensor tendons during proximal phalanx rotation around the metacarpal head.

**FIGURE 7:**
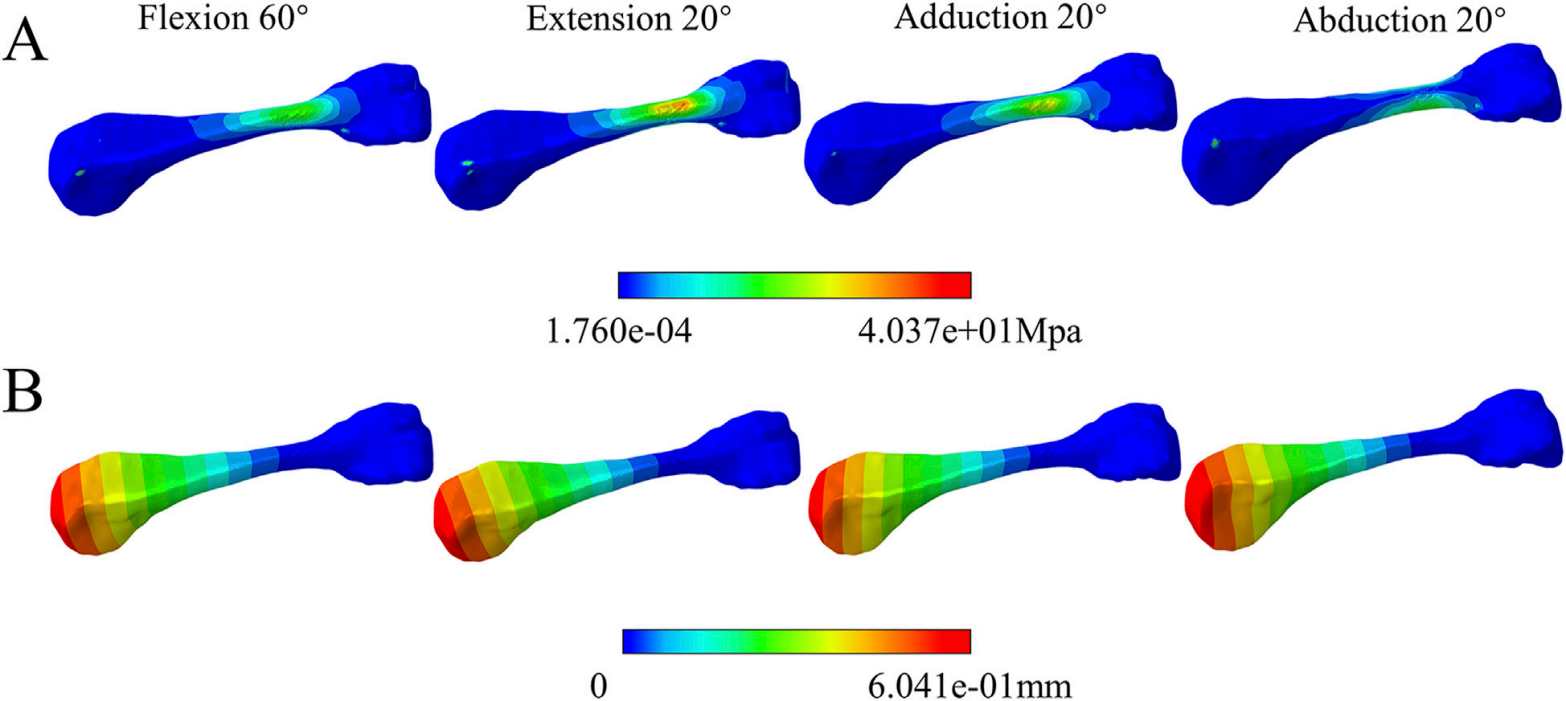
Complete metacarpal component analysis. **(A)** stress cloud maps under various motion states. **(B)** displacement cloud maps under various motion states.

During the loading process of the complete metacarpal, we can consider the applied force to consist of two main components. Firstly, the resultant force is caused by friction and contact stresses generated by the extensor tendon along the *y*-axis of the coordinate system during movement (Figure 6). We observed that the resultant force increased parabolically during flexion by 60° and extension by 20°. At 60° of flexion, the peak resultant force was 2.24 N, while at 20° of extension, the peak resultant force was 0.97 N. There was no influence on the resultant force due to contact during adduction and abduction. Secondly, the rotational forces exerted on the metacarpal by the radial and ulnar collateral ligaments, increased proportionally with the rotation angle (Figure 7A). The stress distribution of the metacarpal under loading conditions is shown, with different colours representing different scenarios (red indicating high stress or displacement areas, blue indicating low stress or displacement areas). The maximum peak stress occurred at 60° of flexion, reaching 40.37 MPa, while the minimum stress at 20° of extension was 7.102 MPa. In descending order, the stress levels were: flexion 60° > abduction20° > adduction 20° > extension 20°. Stress on the metacarpal mainly concentrates near the base of the metacarpal. The displacement distribution of the metacarpal under loading conditions is shown (Figure 7B). Displacement gradually decreased from the distal end to the metacarpal base, with the peak displacement occurring at 60° of flexion, at 0.6041 mm, and the minimum at 20° of extension, at 0.08934 mm. In descending order: flexion 60° > abduction 20° > adduction 20° > extension 20°.

### 3.3 Comparison of peak von mises stress and distribution for four fixation methods

**FIGURE 8:**
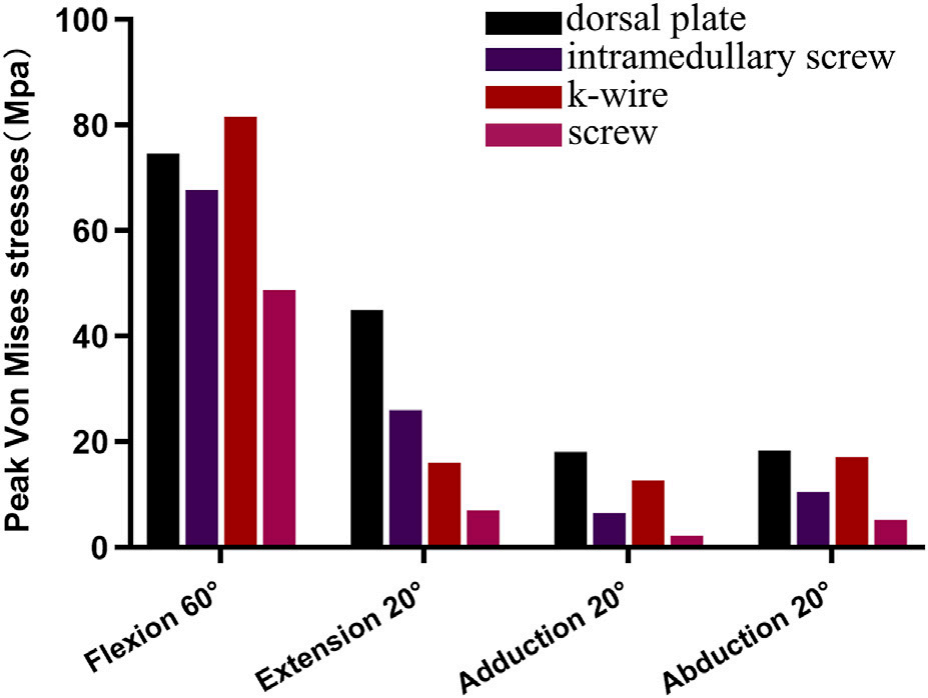
The comparison of peak Von Mises stresses during flexion 60°, extended 20°, adducted 20°, and abducted 20° movements among the various fixtures.

**FIGURE 9:**
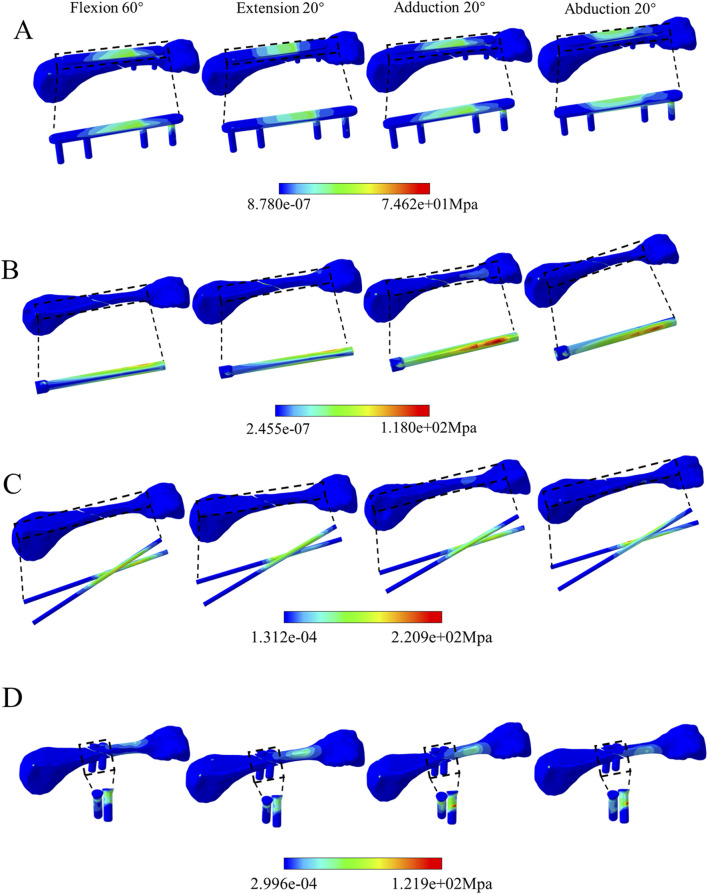
Comparison of Von Mises stress distribution among the four fixation methods. **(A)** Dorsal plate. **(B)** intramedullary screw. **(C)** K-wire. **(D)** screw.

(Table 2) lists the Von Mises stress peaks during proximal phalanx movement around the metacarpal head, and (Figure 8) illustrates the comparison of peak stresses for each fixation device. The dorsal plate fixation exhibited higher peak stresses than other implant groups during extension, adduction, and abduction movements. The Kirschner pin implants showed the highest peak stress of 81.6 Mpa at 60° of flexion compared to the other groups. In contrast, screw implants showed the lowest Von Mises stress peaks among all groups (Figure 9). shows the stress distribution patterns. Stress concentrations for the dorsal plate, intramedullary nail, and Kirschner wire fixations primarily occurred within the implant structures, with no significant stress concentration observed within the metacarpal itself. However, stress in the screw group is still mainly concentrated near the proximal end of the metacarpal close to the base. Specifically, stress concentration in the dorsal plate group occurred near the fracture line, while for the intramedullary nail group, during flexion and extension movements, stress concentrated on the upper and lower surfaces of the nail. During adduction and abduction movements, it concentrated on the lateral surfaces, with higher stress concentration areas near the proximal end of the metacarpal. Stress in the Kirschner wire group mainly concentrates near the fracture line and the proximal end of the metacarpal. Stress on the screws themselves in the screw group was relatively low.

**TABLE 2:** The peak Von Mises stress (MPa) at the distal metacarpal, proximal metacarpal, and fixtures are in various activity statuses and fixtures.

Activity status and fixtures	Distal metacarpal	Proximal metacarpal	Fixtures
Flexion 60°			
Dorsal plate	1.86E+01	3.13E+01	7.46E+01
Intramedullary screw	1.18E+02	1.42E+01	6.78E+01
K-wire	2.21E+02	8.99E+01	8.16E+01
Screw	9.14E+01	1.22E+02	4.88E+01
Extended 20°			
Dorsal plate	1.289	1.04E+01	4.50E+01
Intramedullary screw	7.45	6.709	2.60E+01
K-wire	2.71E+01	1.49E+01	1.60E+01
Screw	1.84E+01	1.30E+01	7.07
Adducted 20°			
Dorsal plate	1.74	1.37E+01	1.81E+01
Intramedullary screw	7.331	2.75	6.552
K-wire	2.25E+01	1.85E+01	1.27E+01
Screw	9.11	4.426	2.321
Abducted 20°			
Dorsal plate	2.398	5.581	1.84E+01
Intramedullary screw	2.65E+01	4.95	1.14E+01
K-wire	2.36E+01	2.05E+01	1.71E+01
Screw	2.30E+01	2.04E+01	5.222

### 3.4 Comparison of peak displacements and distribution for four fixation methods

**FIGURE 10:**
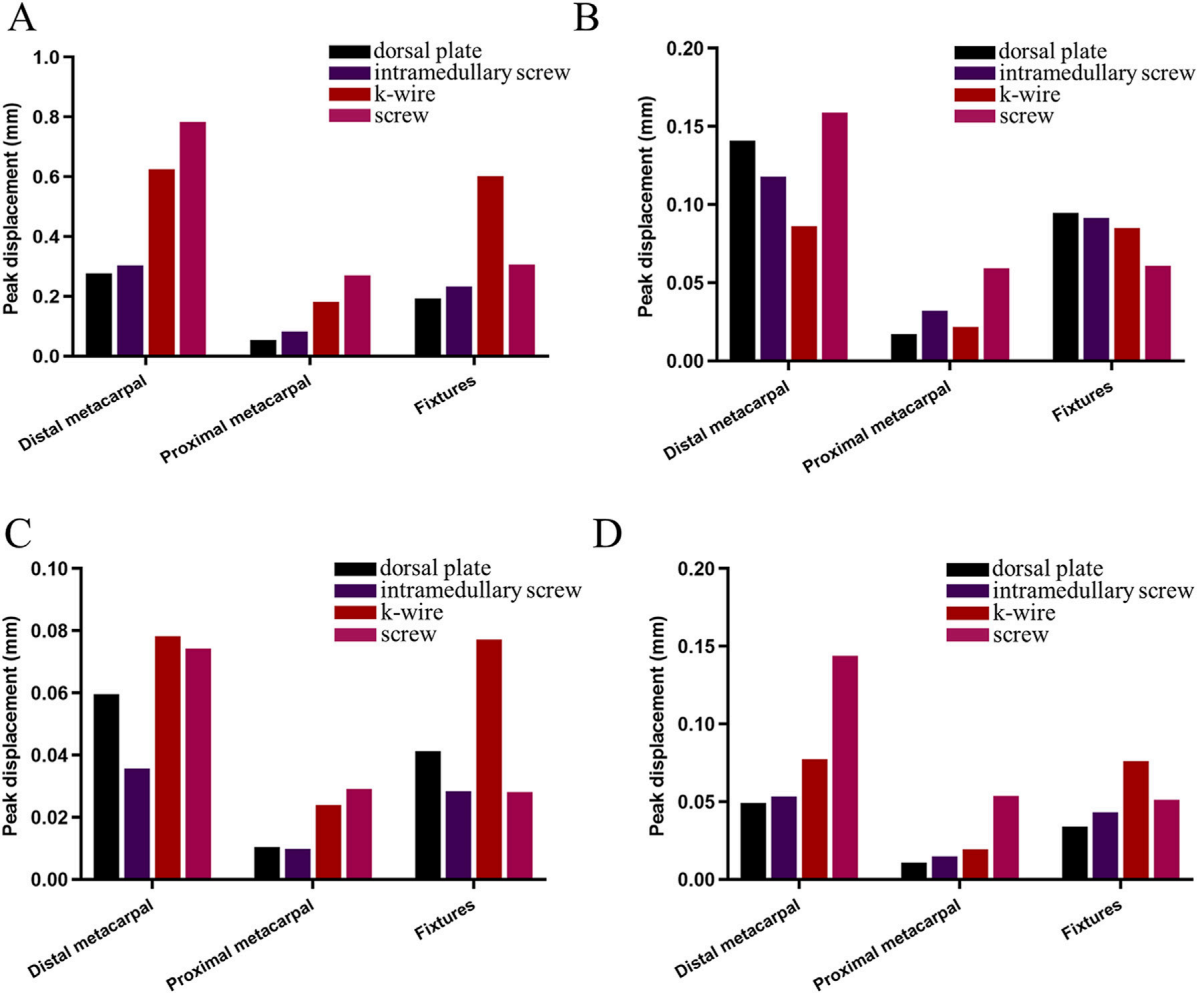
Comparison of peak displacements of the distal metacarpal, proximal metacarpal, and fixtures under various fixation methods during proximal phalanx rotation around the metacarpal head. **(A)** flexion 60°. **(B)** extended 20°. **(C)** adducted 20°. **(D)** abducted 20°.

**FIGURE 11:**
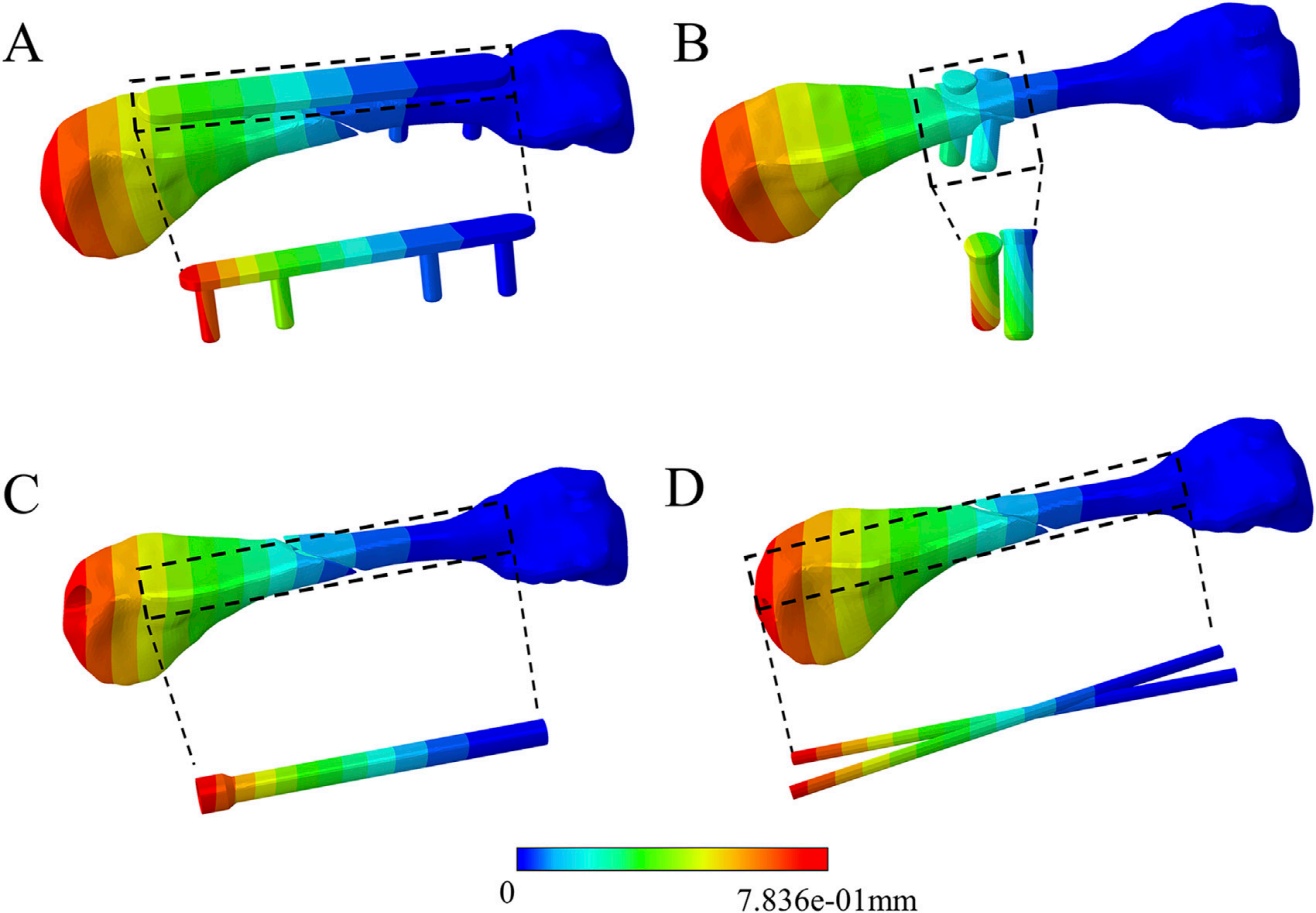
Comparison of displacement distribution among the four fixation methods during 60° flexion of the proximal phalanx. **(A)** dorsal plate. **(B)** Screw. **(C)** Intramedullary screw. **(D)** K-wire.

(Table 3) lists the peak displacements of the distal metacarpal, proximal metacarpal, and implants under various fixation methods during proximal phalanx movement around the metacarpal head. At the same time (Figure 10) illustrates the comparison results. In all displacement tests, the dorsal plate fixation showed maximum stability at 60° of flexion and 20° of abduction, but not at 20° of extension and 20° of adduction, with a peak displacement of 0.19 mm. The peak displacements in the intramedullary nail group were similar to those in the dorsal plate group, with a maximum displacement difference of 0.04 mm, and its stability surpassed that of the dorsal plate during adduction movements. The peak displacements in the Kirschner wire group were relatively unstable, showing lower peak displacements during extension by 20° and larger displacements during adduction by 20°. The peak displacements in the screw group were relatively stable but larger. Regarding displacement distribution, the trend was consistent across all fixation groups, gradually decreasing from the metacarpal head to the base (Figure 11).

**TABLE 3:** The peak displacements (mm) at the distal, proximal metacarpal, and fixtures are in various activity statuses and fixtures.

Activity status and fixtures	Distal metacarpal	Proximal metacarpal	Fixtures
Flexion 60°			
Dorsal plate	2.77E-01	5.47E-02	1.93E-01
Intramedullary screw	3.04E-01	8.31E-02	2.33E-01
K-wire	6.25E-01	1.82E-01	6.02E-01
Screw	7.84E-01	2.70E-01	3.06E-01
Extended 20°			
Dorsal plate	1.41E-01	1.73E-02	9.48E-02
Intramedullary screw	1.18E-01	3.21E-02	9.16E-02
K-wire	8.63E-02	2.18E-02	8.51E-02
Screw	1.59E-01	5.92E-02	6.09E-02
Adducted 20°			
Dorsal plate	5.96E-02	1.05E-02	4.13E-02
Intramedullary screw	3.57E-02	9.89E-03	2.84E-02
K-wire	7.83E-02	2.40E-02	7.72E-02
Screw	7.43E-02	2.91E-02	2.81E-02
Abducted 20°			
Dorsal plate	4.93E-02	1.09E-02	3.41E-02
Intramedullary screw	5.35E-02	1.49E-02	4.33E-02
K-wire	7.75E-02	1.95E-02	7.61E-02
Screw	1.44E-01	5.37E-02	5.12E-02

## 4 Discussion

Surgical treatment of metacarpal fractures requires comprehensive decision-making based on fracture characteristics, soft tissue damage, and the individual functional needs of the patient, and the choice of appropriate fixation is critical (Beck et al., 2019). This is because it needs to provide sufficient stability to facilitate early return to activity (Milz et al., 1999), prevent joint stiffness and tendon adhesion, and ensure normal patient function. Previous studies have relied on cadaveric bones, animal bones, or artificial bones to study the biomechanical stability of metacarpal fractures due to the difficulty of obtaining fresh human metacarpal bones. In addition, many researchers simulated metacarpal fractures by finite element analysis. However, they only applied external loads for static studies, failing to consider the stability differences between different implant fixation methods during simulated finger movements. Therefore, this study investigated the biomechanical characteristics of different implant fixations for oblique metacarpal shaft fractures during simulated proximal phalanx movement around the metacarpal head.

Finite element analysis can illustrate the overall structure’s stress and strain patterns but necessitates considering multiple assumptions and simplifications, such as model, materials, and boundary conditions. Our model involves proximal phalanx movement around the fourth metacarpal head, assuming that the extensor tendons exert contact pressure on the metacarpal head. The complex interaction between the extensor tendons and the metacarpal head influences finger flexibility and stability. Even without specific experimental data and parameters, anatomical structures can infer this possibility. The extensor tendons of the fingers travel straight when extended but wrap around the phalanges during flexion (Milz et al., 1999), with deep fibres merging with the distal aspect of the palmar digital joint capsule and extending to the base of the proximal phalanx (Von Schroeder and Botte, 1995). This layout suggests that the extensor tendons may contact the metacarpal head through the joint capsule. Although this contact is indirect, it may exert certain indirect pressure during flexion. Various factors influence this pressure, including finger movement speed, force, lubrication conditions, and tissue surface roughness. As shown in (Figure 6), during proximal phalanx movement around the metacarpal head at 60° of flexion, we observed the resultant force of contact pressure and friction pressure on the metacarpal head to be 2.24 N. This force value is relatively smaller compared to the forces observed by Butz et al. (2012) in everyday activities at the metacarpophalangeal joint, such as keyboard typing (11.3 N), writing with a pen (20.1 N), and playing the piano (31.6 N). This indicates that our model simulated finger movement without external loads, indirectly validating its relative effectiveness. Furthermore, our model also considered the contribution of collateral ligaments on the radial and ulnar sides to the stability of the metacarpophalangeal joint. These ligaments extend obliquely from the metacarpal head to the proximal phalanx (Schultz et al., 1987), contributing to the stability of the metacarpophalangeal joint.

In this study, we conducted a finite element analysis to simulate the biomechanical performance of four fixation methods for oblique metacarpal shaft fractures under different finger motion states. Below is a detailed biomechanical analysis of each fixation method and a discussion of its clinical significance.

Plate fixation exhibited higher stability and minimal displacement when the fingers were flexed at 60° and abducted at 20°. However, the stress levels with plate fixation were consistently higher in all motion states, especially near the fracture line and the proximal aspect of the metacarpal. This suggests that while plates offer good mechanical stability, the risk of implant failure may increase due to stress concentration. Additionally, friction between the plate and extensor tendons might contribute to the elevated stress. Clinically, plate fixation is suitable for cases requiring high stability, such as severe displacement or complex comminuted fractures. This fixation method allows for reliable stability, enabling early mobilisation and engagement in strenuous activities (Dreyfuss et al., 2019). However, we should exercise caution to avoid overloading, which could lead to implant failure. We should prevent complications such as tendon irritation or rupture due to friction between the plate and tendons (Xu et al., 2021).

Intramedullary nail fixation exhibited relatively uniform stress distribution across all motion states, with lower stress levels than plate fixation, indicating a lower risk of failure. It provided stability in various motion states, demonstrating superior stability to plates when the fingers experienced adduction at 20°. Clinically, intramedullary nail fixation is suitable for patients requiring a rapid return to daily activities, particularly male patients of working age (Beck et al., 2019). However, it is essential to acknowledge the inherent risks of tendon and articular cartilage damage associated with intramedullary fixation. Further clinical studies are needed to elucidate the long-term clinical outcomes of intramedullary nail fixation.

Kirschner wires exhibited the highest stress levels and instability when flexing the fingers at 60°, indicating a propensity for failure. Clinically, K-wire fixation is cost-effective and minimally invasive, suitable for simple fractures or patients with limited economic resources. However, close monitoring is required to mitigate risks such as pin tract infection and structural migration (Ruchelsman et al., 2014). Increasing the diameter of Kirschner wires or combining them with plate fixation can enhance stability (Zhang et al., 2015; Zhu et al., 2017; Amsallem et al., 2018).

Screws demonstrated lower stress levels across all finger motion states, with the lowest risk of failure, albeit with poorer stability. Screw fixation is suitable for cases not requiring high mechanical stability, such as mild fractures, elderly patients, or those not subjected to significant physical impact post-surgery. Due to its lower stress levels, screw fixation carries a lower risk of failure but requires attention to its inadequate stability.

In our study, the stress distribution in the plate group aligns with the previous findings by Zhang et al. (2022), indicating that stress primarily concentrates in the plate area near the fracture line. Additionally, Galbraith et al. (2022) found in their research that in terms of cantilever bending and torsion, intramedullary nails demonstrate superior biomechanical performance compared to Kirschner wires. However, their stability is less robust than that of plates. In our investigation, through simulating finger motion states, we discovered that intramedullary nails exhibit better performance relative to Kirschner wires in flexion, internal rotation, and abduction states. Although intramedullary nails generally exhibit lower stability than plates, their stability surpasses that of plates at 20° of adduction. Chiu et al. (2022) found that compared to plate fixation, double screw fixation exhibits a slightly lower maximum fracture load but similar stiffness. In this study, double screw fixation demonstrates a lower risk of failure compared to plate fixation, albeit with less stability. Our study further expands the understanding of implant stability for metacarpal fractures regarding dynamic performance, providing a more comprehensive basis for the clinical selection of the optimal fixation method.

Through the detailed biomechanical analysis and clinical application discussion above, we aim to provide clinicians with more precise treatment options to guide early rehabilitation and minimise the risk of treatment complications, improving patient outcomes. However, this study has some limitations. For instance, the complexity of finger joint motion, simplifying assumptions in the model, neglect of screw preload, and material property simplifications may affect the accuracy of the model and the generalizability of experimental results. Future research should consider more complex models, including interactions of multiple muscles, dynamic loading conditions, and more realistic material properties. Additionally, the design of clinical trials should consider the findings of these biomechanical studies to validate the efficacy of fixation methods and optimise treatment strategies.

## 5 Conclusion

This study utilised finite element analysis to simulate the biomechanical effects of four fixation methods for oblique metacarpal shaft fractures under various finger motion states. The findings indicate that plate fixation provides the highest stability, particularly during finger flexion and abduction, albeit with the highest stress levels, increasing the risk of failure. Intramedullary nail fixation demonstrates similar stability to plates in most motion states but with lower stress levels, reducing the risk of failure. Although Kirschner wire fixation is cost-effective and minimally invasive, it is prone to failure during flexion and exhibits unstable displacement. Despite its weaker stability, screw fixation carries the lowest risk of failure due to lower stress levels. These findings offer vital guidance for clinicians in selecting fixation methods, making more precise treatment decisions based on individual patient characteristics and fracture types, promoting early rehabilitation and reducing the risk of complications.

## Data Availability

The original contributions presented in the study are included in the article/supplementary material, further inquiries can be directed to the corresponding authors.
